# Bone-Derived Extracellular Vesicles: Novel Players of Interorgan Crosstalk

**DOI:** 10.3389/fendo.2019.00846

**Published:** 2019-12-10

**Authors:** Yi Li, Pengbin Yin, Zhongkui Guo, Houchen Lv, Yuan Deng, Ming Chen, Ya Gu, Peifu Tang, Licheng Zhang

**Affiliations:** Department of Orthopedics, General Hospital of Chinese PLA, Beijing, China

**Keywords:** extracellular vesicles, interorgan crosstalk, bone factors, osteocalcin, FGF23 = fibroblast growth factor 23, exosomes

## Abstract

An increasing number of studies have shown that bone plays an active role in regulating glucose metabolism, affects renal, and cardiovascular diseases and even influences the development of offspring. These novel findings have indicated that bone plays a much more important role in the human body than only providing physical support. However, further investigations of the mechanisms underlying the effects of bone are needed. Recently, extracellular vesicles (EVs) have received increased attention because they can transfer functional proteins, mRNAs, and miRNAs between cells/organs. After reviewing the existing evidence, we hypothesized that bone may be involved in interorgan communication via EVs. Further research exploring bone-derived EVs may facilitate the understanding of bone as a multifunctional organ.

## Introduction

As research has progressed, the functions of various organs have been updated. The common function of bone is traditionally believed to be that of an effector. Bone is usually a target regulated by other organs for physiological function, and to an extent, bone acts as a recipient during physiological communication. However, recent studies have proven that bone not only acts as an effector but also regulates other organs by secreting biological molecules. For example, fibroblast growth factor 23 (FGF23) secreted from osteoblasts and osteocytes can regulate phosphate and mineral metabolism (1, 2). Bone-derived osteocalcin (OCN) and lipocalin-2 (LCN2) regulate glucose metabolism and improve insulin resistance. In spite of its metabolic functions, OCN could cross the blood-brain barrier and promote learning and memory (3–5). Receptor activator of NF-κB ligand (RANKL) and its receptor Receptor activator of NF-kB (RANK) are essential regulators of bone remodeling. Studies have shown that RANKL and RANK are also expressed in the central nervous system and have unexpected functions in controlling inflammation in ischemic brains (6, 7). These results strongly suggest that bone has more functions that were ignored by previous research. Bone may regulate the function of other organs under physiological conditions. However, thus far, this novel function of bone has not been fully clarified, as studies generally focus on specific proteins. Few new factors were found to further reveal the characteristic features of bone. The mechanisms underlying these regulatory functions remain a mystery.

Recent studies have found that various organ systems can exchange information between cells through extracellular vesicles (EVs), which is a new mechanism of communication between cells and organs. Studies have found that EVs from different sources are widely involved in endocrine, tumor microenvironment, and nervous system regulation (8–10). These findings indicate that EVs could provide a means for remote tissue communication. Although few studies have found that bone-derived EVs can participate in interorgan communication, the role of EVs involved in intercellular communication during bone metabolism has been widely discovered. EVs have been identified as having important functions in regulating the communication between osteoblasts and osteoclasts. The most important discovery is that osteoclast-derived exosomal miR-214-3p could be transferred to osteoblasts to inhibit osteoblast activity (11, 12). Studies have shown that osteoblast-derived EVs could also contain mRNAs that contribute to the RANKL pro-osteoclastic effects (13). Since bone-related EVs have been found to be important bioactive components involved in bone regulation, are bone-related EVs involved in interorgan regulation?

## Hypothesis

Recent studies have found that bone could affect multiple organs and be involved in physiological and pathological changes by secreting biomolecules (14, 15). Notably, EVs, 30–1,000 nm in diameter, which include exosomes and microvesicles, have been demonstrated to transfer functional proteins, mRNAs, and miRNAs to neighboring cells and serve as mediators of intercellular and long-distance communication (9, 16–18). Researchers have found that EVs could participate in interorgan communication and play an important role in physiological regulation. In addition, it was found that bone-related cells can also exchange genetic information and regulate bone remodeling through EVs (19, 20). Given these findings, we hypothesized that bone-related EVs may represent an important paradigm for remote organ effects.

## Evaluation of the Hypothesis

### EVs Participate in Interorgan Communication

With the deepening of research, investigators have paid attention to EVs as biomarkers in the early stage and have gradually discovered that EVs can stably transfer proteins and genetic material, which suggests that EVs may play a potential role in physiological regulation.

More importantly, the discovery of circulating EVs has given researchers new inspiration. Fruhbeis et al. found that exercise triggered a rapid release of EVs into the circulation. The dynamics of EVs varied between cycling and running exercise. The results suggest that EVs released during physical activity may participate in long-distance signaling during exercise-mediated adaptation processes (21). Guescini et al. found that muscle tissue could release EVs carrying muscle-specific miR-133b and miR-181a-5p under regular physical exercise (22). Thomou et al. determined that adipose tissue constitutes an important source of circulating exosomal miRNAs, which can regulate the expression of fibroblast growth factor 21 (FGF21) in liver tissues. These results indicate that adipose-derived exosomal miRNAs can regulate distant organs (23). Ying et al. also found that macrophages in adipose tissue secreted EVs, which could transfer miRNAs to insulin target cells. These EVs can modulate systemic insulin and glucose tolerance by directly affecting cellular insulin signaling (24). In addition to genetic materials, circulating exosomal proteins also have potential functions. Whitham et al. found that EVs liberated by exercise have a propensity to localize in the liver and can transfer their protein cargo (25). These results provide evidence for a new paradigm, namely, EV trafficking, which can act as a bridge in interorgan communication and exert systemic biological effects. More interestingly, Yoshida et al. found that EVs containing eNAMPT can delay aging and prolong life in mice. They also confirmed that eNAMPT in human plasma is mainly contained in EVs (26).

In addition to participating in energy metabolism, circulating exosomes have also been found to be widely involved in the physiological and pathological processes of the heart. Cheng et al. found that circulating myo-miRs are carried in exosomes and mediate functional crosstalk between the ischemic heart and the bone marrow (BM). They found a higher level of circulating exosomes in acute myocardial infarction (AMI) mice that could transfer myo-miRs into BM monocular cells (MNCs). The results also showed that AMI exosomes could mediate the downregulation of CXC chemokine receptor 4 (CXCR4) and contribute to BM progenitor cell mobilization (27). However, the function of circulating exosomes has not been entirely elucidated. These studies suggest that EVs transferring genetic cargos or proteins could constitute a previously undescribed class of regulators that control metabolism in distant tissues, providing a new mechanism of cell–cell crosstalk. Based on the above evidence, it is suggested that EV trafficking may be an important biological process of interorgan communication.

## Bone-Secreted Factors Participate in Interorgan Communication

**Figure 1:**
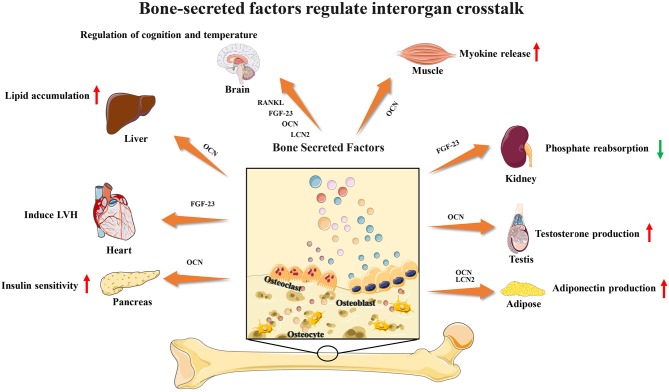
Bone-secreted factors participate in interorgan communication.

Recent studies have found that several bone-secreted factors participate in interorgan communication (Figure 1). Osteocalcin is an osteoblast-specific protein that is secreted at high levels in the bone extracellular matrix, and the genes encoding osteocalcin start to be expressed during development around the time bone mineralization begins (15). Many studies have shown that OCN plays a key role in the regulation of energy metabolism. For adipose tissue, OCN treatment could upregulate the expression of adiponectin, improve glucose uptake and insulin sensitivity *in vivo* and suppress the secretion of proinflammatory cytokines in adipocytes *in vitro* (28). OCN supports muscle function during exercise in part through the release of IL-6, the first myokine found to be rapidly released into the blood during exercise, enhancing glucose and fatty acid uptake into myofibers (29, 30). These observations and studies suggest that the effects of OCN on obesity and insulin resistance could be a result of its capacity to promote insulin sensitivity in the liver and adipose tissue, energy expenditure in muscle and insulin production in the pancreas and to upregulate expression of functional genes in the pancreas, muscle and adipose tissue. Male osteocalcin^−/−^ mice had low circulating levels of testosterone and bred poorly, which indicated that OCN may also regulate reproductive functions (5). Moreover, many unexpected observations showed that osteocalcin may influence the development of the brain and cognitive functions of the offspring. Osteocalcin is necessary and sufficient to correct age-related declines in cognitive function in mice. Intriguingly, the maternal production of osteocalcin appears to be necessary for normal fetal brain development (5, 31, 32).

FGF23 is a phosphaturic hormone produced by osteocytes and osteoblasts. FGF23 mainly targets the renal proximal tubules to inhibit calcitriol production and the expression of the sodium/phosphate cotransporters NaPi2a and NaPi2c, thus inhibiting renal phosphate reabsorption (1, 2, 33). Furthermore, recent studies have found that the increased level of FGF23 may induce left ventricular hypertrophy (LVH) and is associated with a risk of mortality (34). However, several large epidemiological studies demonstrated a powerful dose-dependent association between serum levels of FGF23 and a higher risk of mortality in end-stage renal disease (ESRD) patients (35–37). These observations showed that FGF23 may connect bone with heart diseases and renal disorders. In addition to these two factors, bone-derived lipocalin2 (LCN2) was also found to suppress appetite and regulate fat mass, which indicated a potential connection between bone and brain (38, 39).

In recent years, researchers have found that RANK neurons are involved in the regulation of body temperature in the preoptic area of the hypothalamus. In stroke, the RANKL-RANK signaling pathway protects neurons and reduces nerve damage. In autoimmune diseases, RANKL can participate in and assist Th17 cells in entering the brain parenchyma through the blood-brain barrier. In metabolic regulation, RANK signaling is involved in modulating Neuropeptide Y(NPY) levels and through that matching bone mass to body weight. The selective deletion of RANK from NPY neurons leads to a significant increase in fat mass and a decrease in whole body bone mineral density (6, 7, 40–42).

As more and more bone-secreted factors were confirmed to be involved in interorgan communication, we can conclude that bone may actively participate in the regulation of physiological processes.

## Bone-Secreted Factors Transferred by EVs

However, there is no definitive evidence to date that bone-derived EVs could regulate remote organs. Various studies have found that bone secreted factors involved in interorgan communication could be transferred through EVs. Yi et al. found that OCN could be packed into exosomes and is able to be transferred to the aorta endothelial cells via exosome incorporation (43). Previous studies have indicated that the serum level of OCN is inversely associated with atherosclerosis. However, the expression of OCN in endothelial progenitor cells (EPCs) has not been clarified. Importantly, this study indicated that OCN-exosomes could promote the proliferation of endothelial cells efficiently via OCN-GPRC6A signaling. In addition, LCN2 was also found to be packed in EVs. Rollet-Cohen et al. compared the proteomic content of respiratory exosomes from cystic fibrosis, primary ciliary dyskinesia and asthma patients. The results showed high expression of LCN2 in exosomes from cystic fibrosis patients (44). The above studies suggest that EVs may contain bone-derived factors and play a regulatory role in physiological or pathological conditions. Because current research has not elucidated the complete function of bone-derived EVs, further research should focus on the relationship between EVs and bone-derived factors.

## Paracrine Role of Bone-Derived EVs

Bone is a dynamic organ that is constantly remodeled by the activities of several cells, including mesenchymal stem cells, osteoblasts, osteocytes, and osteoclasts. Many studies have found that bone-derived EVs play important roles in cellular communication in bone remodeling. In the bone-remodeling microenvironment, bone-derived EVs contain specific proteins and genetic material and are involved in regulating cell activity.

Bidirectional osteoblast-osteoclast communication plays crucial roles in bone remodeling (45). Cappariello et al. found that osteoblast-derived EVs are involved in intercellular communication and facilitate the function of osteoclasts. In osteoblasts pretreated with parathyroid hormone (PTH), EVs from osteoblasts contain RANKL in their outer membrane and are captured by osteoclasts. More importantly, PTH also increased the total number of RANKL-positive EVs. In addition, this study also showed that EVs are biotechnological tools to shuttle antiosteoclastic drugs such as dasatinib and zoledronate. To investigate the role of osteoblast-derived EVs, they used the RANKL^−/−^ mice, a murine model characterized by the lack of osteoclasts. Osteoclasts in RANKL^−/−^ mice were inactive due to the lack of pro-osteoclastic function. The osteoblast-derived EVs stimulated osteoclasts in RANKL^/−^ mice. Osteoblast-derived EVs could recover this function by inducing TRAcp-positive cells (46, 47). On the other hand, osteoclast-derived EVs can also affect osteoblast function. Li et al. and Sun et al. showed that exosomal miR-214-3p from osteoclasts, which participated in the crosstalk of bone cells, inhibited osteoblast activity and reduced bone formation (11, 12). Mesenchymal stem cells (MSCs) are multipotent cells that can differentiate into bone-related cells. Therefore, MSCs are often used as seed cells for bone repair (48–51). However, the paracrine factors of MSCs, rather than exogenous MSCs themselves, were recently shown to play a major role in promoting tissue repair (52, 53). Recent studies have revealed that EVs derived from MSCs could transfer genetic material for cell communication, thereby regulating the main processes of bone regeneration, including osteogenesis and angiogenesis (19). Qin et al. found that MSC-derived EVs were endocytosed by osteoblasts and regulated their activity *in vitro* (54). MSC-derived EVs could also be captured by human umbilical-vein endothelial cells (HUVECs) and transmit miR-494 to stimulate angiogenesis *in vitro* and *in vivo* (55). Likewise, endothelial cell-derived EVs could be taken up by MSCs and transmit miR-31 to inhibit osteogenic differentiation (56).

Sato et al. compared osteocyte-secreted exosomes and circulating exosomes from osteocyte-less mice. The results indicated that ablation of osteocytes in mice alters the miRNA levels of plasma exosomes (57). This study linked osteocyte exosomes to circulating exosomes, which provides the concept that osteocyte-derived exosomes may transfer their components to other organs.

In conclusion, paracrine functions of bone-derived EVs are widely involved in intercellular communication and genetic material transmission in the bone microenvironment.

## Major Questions Remain in Verifying this Hypothesis

There are still many key issues that need to be addressed to prove this hypothesis. First, current research has confirmed that bone secreted factors play a role in the regulation of brain, liver, muscle, pancreas, testis, and adipose tissue. Further research should begin with these known directions to explore whether bone-related EVs are involved in the regulation of these organs. Second, it is also important to identify the EV-mediated bone effects on the physiological functions of other organs. Current research has primarily explored the role of bone in endocrine and energy metabolism, but whether bone has effects on other systems, such as the immune system, requires further exploration.

Another important problem is the lack of knowledge of bone-derived EVs. Most studies published thus far have analyzed mixed EV populations, and the most important step is to comprehensively compare the different subtypes of EVs (58). Furthermore, specific markers or characteristics of EVs from different cells are also needed for identification. To determine the roles of bone-derived EVs in interorgan communication, researchers need to identify the subtypes and characteristics of EVs from bone-related cells and determine whether different subtypes have specific functions or even prominent target organs. Furthermore, important questions remain regarding the spatiotemporal properties of EVs *in vivo* (18). Currently, many studies have shown the effects of EVs on target cells, but most studies are performed *in vitro* or *in vivo* by injection of EVs harvested from cells in culture. The characteristics of bone-derived EVs in physiological states require further exploration. Further study should illuminate the life span, the concentration and the *in vivo* metabolism of bone-derived EVs in the bloodstream. As a result, these studies may not reflect the spatiotemporal properties or concentrations of EVs active in normal physiology *in vivo*. A direct demonstration that functional EV-mediated biological cargo transfer is the relevant mechanism in certain biological processes is still difficult to achieve (59).

## Importance of Confirming this Hypothesis

Confirmation of EV-mediated interorgan communication of bone would help deepen the understanding of the communication networks between the skeleton and other systems. The results of existing research have shown that bone-secreted proteins could regulate interorgan communication. The growing awareness that bone is an active organ will broaden our understanding of the pathogenesis of bone in other systemic diseases. The interorgan communication function of bone mediated by EVs may become a therapeutic target for other diseases.

Furthermore, confirming this hypothesis may expand our knowledge of EVs. Many studies have confirmed the widespread presence of EVs in body fluids. However, the biological functions of biofluid-derived EVs remain unclear. The current study mainly focused on EVs from body fluids for disease diagnosis, but their physiological functions still need to be revealed. In addition, the role of EVs is not only transferring functional proteins but also transferring genetic material. Confirmation of the regulation of bone-secreted EVs on multiple organs will help us better understand the transmission of genetic information. Meanwhile, as EVs have been acknowledged as natural targeted-drug carriers, EV-based nanotechnology has provided unprecedented opportunities to help develop EV-related therapeutics (60, 61). Research into the communication between skeleton-related EVs and other organs may identify novel therapeutic targets for medical intervention.

## Author Contributions

LZ and PT made major contributions to the conception of the work. YL and PY drafted the manuscript. ZG, MC, HL,YD, and YG revised the manuscript.

### Conflict of Interest

The authors declare that the research was conducted in the absence of any commercial or financial relationships that could be construed as a potential conflict of interest.
